# Software infrastructure and data pipelines established for technical interoperability within a cross-border cooperation for the flora of the Bohemian Forest

**DOI:** 10.3897/BDJ.10.e87254

**Published:** 2022-10-14

**Authors:** Petr Novotný, Stefan Seifert, Martin Rohn, Wolfgang Diewald, Milan Štech, Dagmar Triebel

**Affiliations:** 1 Department of Biology Education, Faculty of Science, Charles University, Praha, Czech Republic Department of Biology Education, Faculty of Science, Charles University Praha Czech Republic; 2 Staatliche Naturwissenschaftliche Sammlungen Bayerns, SNSB IT Center, Munich, Germany, Munich, Germany Staatliche Naturwissenschaftliche Sammlungen Bayerns, SNSB IT Center, Munich, Germany Munich Germany; 3 Babická 2379/1a, Praha, Czech Republic Babická 2379/1a Praha Czech Republic; 4 Staatliche Naturwissenschaftliche Sammlungen Bayerns, Botanische Staatssammlung München, Munich, Germany Staatliche Naturwissenschaftliche Sammlungen Bayerns, Botanische Staatssammlung München Munich Germany; 5 Department of Botany, Faculty of Science, University of South Bohemia, České Budějovice, Czech Republic Department of Botany, Faculty of Science, University of South Bohemia České Budějovice Czech Republic

**Keywords:** Flora Silvae Gabretae, Bohemian Forest, occurrence data portal, ABCD occurrence data sources, taxon names services, Pladias, web portal, Diversity Workbench repositories.

## Abstract

**Background:**

The timely and geographical resolutions, as well as the quantity and taxon concepts of records on the occurrence of plants near national borders is often ambiguous. This is due to the regional focus and different approaches of the contributing national and regional databases and networks of the neighbouring countries. Careful data transformation between national data providers is essential for understanding distribution patterns and its dynamics for organisms in areas along the national borders. Sharing occurrence data through the international data aggregator Global Biodiversity Information Facility (GBIF) is also complicated and has to consider that the underlying taxonomic concept and geographic information system of each single GBIF dataset might be different. In addition, some regional data providers have a restrictive (non-cc) licensing policy which does not allow data publication via the GBIF network. Therefore, it is necessary to investigate new ways to make data fit for use for a better and comprehensive understanding of the Flora of the Bohemian Forest.

**New information:**

In this paper, we present a bilateral technical interoperability solution for vascular plant occurrence data for the area between the Czech Republic and Bavaria. We describe the initial state of data providers in both countries and the factual and technical challenges in finding a sustainable concept to establish mutual data sharing. The resulting solution for a functional infrastructure and an agreed data pipeline is described in a step-by-step approach. The new distributed infrastructure allows botanists and other stakeholders from both countries to work within the cross-border context of historical and current plants' distribution.

## Introduction

**Figure 1.**
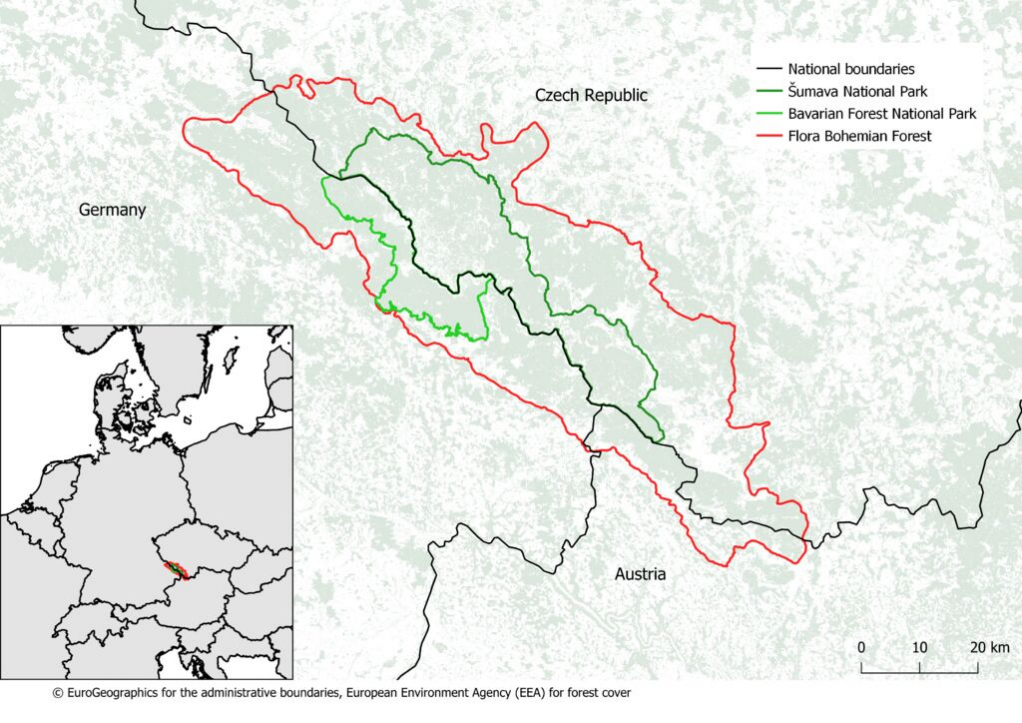
Geographical context of the study area –⁠ Bohemian Forest.

The Bohemian Forest is a biologically extraordinary region in Central Europe (see Fig. 1), attracting significant attention regarding the exploration and documentation of its biodiversity and nature conservation. It is a collective natural heritage of the Czech Republic, Germany (Bavaria) and Austria and has been formed by many natural factors, as well as human influence. Regardless of natural influences, important factors for the various patterns of flora in the region were the diverse types of land-use in different areas across countries and their change over time. Nature conservation issues are mostly handled by the two adjacent national parks situated on opposite sides of the Czech and Bavarian national border with their profound and shared knowledge of the natural heritage. The Austrian region forms the smallest part of study area. Due to organisational reasons (bilateral funding), its data were not included in this stage of cross-border integration.

The research on the flora of the Bohemian Forest (*Flora Silvae Gabretae*, FSG) has a long tradition on both sides of the border (Procházka 2000). However, due to the linguistic and, above all, political boundaries, real joint research did not begin until after the fall of the Iron Curtain (Štech et al. 2021). At the time when the political situation allowed for a smooth development of a cross-border research cooperation, methodological problems began to arise. These were solved within the EU INTERREG A funded project whose results relating to data integration are presented here. The project brought together the available data on occurrence and ecological requirements of vascular plants in the Bohemian Forest – independently from country borders. A critical analysis of these data consequently results in an online presentation of the present and historical distribution, as well as of the occurrence frequency of plant species. Apart from integration on the database level, a general audience trilingual website (Czech, German and English)*^1^ provides distribution maps and ecological information about individual taxa.

As discussed below, it was necessary to establish a consensus (compromise) FSG taxonomic and nomenclatural approach linking the two national databases for such an output. Such output itself is of great importance for further conceptual planning and a crucial source of information for research and policy (Reyserhove et al. 2020). It gives direction for meaningful prioritisation of research with regard to species and ecosystem conservation, including the monitoring of population development of rare and endangered species. However, a correct and operational connection of checklists from different regions with different traditions of taxonomic treatments of critical groups is a real challenge.

**Figure 2.**
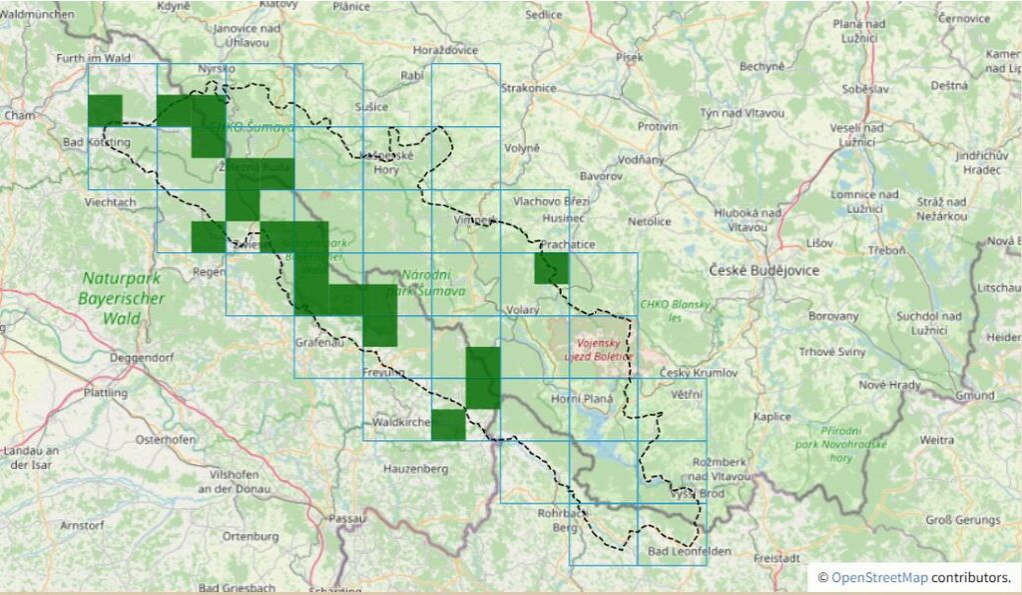
Distribution of *Teucriumscorodonia* according to the joint Czech and Bavarian data. Green rectangles represent grid quadrants (= 1/4 CEBA) with confirmed occurrence, a thick black line demarcates the study area. The frequency of occurrence on the Czech part does not correspond with the information from Bavaria. From https://www.florasilvaegabretae.eu/en/taxon/info/Teucrium%20scorodonia.

**Figure 3.**
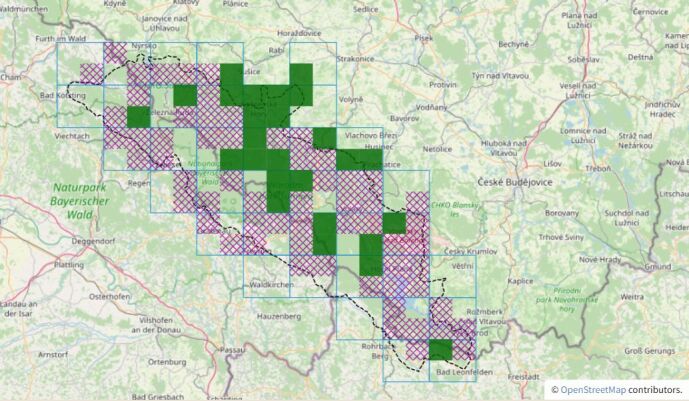
Distribution of *Antennariadioica* according to the joint Czech and Bavarian data. Green rectangles represent grid quadrants (= 1/4 CEBA) with occurrence confirmed after year 2000, purple-hatched squares represent grid quadrants with occurrence confirmed only before 2000. A thick black line demarcates the study area. Patterns of species decline in both countries differ. From https://www.florasilvaegabretae.eu/en/taxon/info/Antennaria%20dioica.

Combining taxon distribution information from both countries and rather the establishment of the software infrastructure and data pipelines helped with the discovery of a number of interesting distribution patterns. Two botanical examples in an otherwise technical article: The occurrence pattern of *Teucriumscorodonia* is completely different on both sides of the border (see Fig. 2) and it will be interesting to obtain more insights into the reasons why by future research. We were also able to identify a different intensity of species decline in both countries, as visible from joint FSG records and date filtered maps (e.g. of *Antennariadioica*, see Fig. 3).

### Cooperation with Pladias

The Czech botanical database Pladias aggregates critically revised vascular plant occurrence data from a dozen local fragmented data providers from the Czech Republic (Wild et al. 2019). In addition to the occurrence data, the platform also contains plant traits, which makes it a very effective tool for botanical syntheses within the Central European region (Chytrý et al. 2021).

The occurrences are limited strictly to the Czech Republic area, allowing only a technical buffer (50 m) across the country border. The platform had been developed with this limitation to store only those data which can be validated and curated by local experts (Novotný et al. 2022).

The Pladias database and system is hosted at the IT Center of the Czech Academy of Sciences, Institute of Botany, Průhonice. The services providing interoperability with Bavarian data are hosted at the University of South Bohemia, České Budějovice.

### Cooperation with the Flora of Bavaria initiative

There are two Bavarian partners involved in the cross-border cooperation, the National Park Bayerischer Wald and the Bavarian Natural History Collections (SNSB). Both partners are strongly involved in the Flora of Bavaria (BFL) initiative, whose data originate from a number of historical and current projects (see history*^2^). In addition, current activities of the SNSB partner have strong relationships with two scientific projects: "*Koordinationsstelle für Florenschutz in Bayern*"*^3^ and "*Aktualisierung der Roten Liste der Gefäßpflanzen Bayerns*"*^4^.

In consequence, the data which are generated, mobilised and quality-controlled as a part of the FSG initiative*^5^ are reused for the Flora of Bavaria initiative and Botanischer Informationsknoten BIB portal*^6^ and vice versa. The technical platform repository is installed at the IT Center of the Bavarian Natural History Collections (SNSB) and the data are generally handled in the BFL instance of the data management platform Diversity Workbench (DWB, see below)*^7^.

## Project description

### Title

Software infrastructure and data pipelines established within a cross-border cooperation for the flora of the Bohemian Forest.

### Study area description

Vascular plant flora of the Bohemian Forest.

The reason to choose the study area was because the Bohemian Forest and its extraordinary flora are regarded as a collective natural heritage of the Czechs and Germans, formed by migration and extinction events of natural as well as human origin. The research on the flora has a long tradition on both sides of the border. A comprehensive survey, however, was challenging as the methodical approaches and the intensity of research in the single subregions varied and the cooperation between the experts was - because of language and political reasons - limited. In consequence, the aim of the project Flora of the Bohemian Forest (= Flora Silvae Gabretae) was to establish a viable integration between two well-known disparate database systems. The two-source database platforms were developed with slightly different ambitions, amongst others, described below. The Czech Pladias is intended for exclusively one group of organisms, i.e. vascular plants, with a checklist codified by the informal authority of administrators. This serves as a protection against "taxon concept inflation" (Kennedy et al. 2006). The Bayernflora (BFL) instance of DWB, on the other hand, relies on several powerful editing tools which are generic and flexible enough to be used for managing and publishing occurrence data from various organism groups, amongst them vascular plants. Unlike Pladias, it allows and actively uses parallel taxon concepts which are administrated separately from the occurrence data in a taxonomy and classification database.

The following is a more detailed description of both partners´databases, data collections and context.

**Area of interest, data sources and calculated data**

The total size of the study area is 2764 km^2^, of which two thirds are the Czech part, one third is the Bavarian part with a small piece of the area belonging to Austria. An overview is given in Table 1. For the floristic mapping, the CEBA (Central European Basic Area) grid system, divided into "quadrants" of 5 × 3 arc minutes, is used (template sensu Niklfeld (1999)). This is 1/4 CEBA, (corresponding to approximately 5.5 × 5.9 km) in both countries.

As of December 2021, there are about 576,673 point-referenced individual occurrence records and 13,833 grid-referenced records from the study area in Pladias. The study area comprises 126 of the regular 2,552 1/4 CEBA topographic Grid raster units in the Czech Republic. These data are aggregated from 11 individual Czech providers, in the vast majority with a reserved licence. There are 1,615 taxa in Pladias with taxon concepts accepted for the study area and agreed amongst the FSG partners (see Table 1).

The individual occurrences have a diverse origin. They are from literature excerpts, as well as records based on revisions of herbarium specimens. All are incorporated in datasets of particular providers. A significant part comes from field observations. The most recent field observations are collected by specialists of critical groups. They build the main proportion of the data under the CC-BY licence. This open licence is newly used in the Pladias database and its usage was started by the recent joint FSG project. We hope that, in the future, significantly more data will be transferred under this licence, thus allowing easier international cooperation and data sharing.

The study area in Bavaria (called Bavarian Forest), comprises 50 of the regular 2,285 topographic Grid raster units in Bavaria. These TK25/4 "quadrants" are equivalent to CEBA quadrants (Petřík et al. 2010, Niklfeld and Wittig 2012). There are two main BFL data sources. Both are aggregated from various sources of provenance from a number of individual Bavarian data providers and local flora projects and are documented accordingly.

The data of the first data source "Flora of Bavaria ─ occurrence data online"*^8^ are categorised as observations, herbarium records/specimens and literature. The majority are grid-based, the minority being GIS referenced and/or with descriptive locality indicated (see Ruff et al. 2019). The FSG occurrences from volunteer experts (currently 237,734 of the 361,173) are an integral part of this BFL data source.

The other main source called "Floristic records from survey studies of the Bayerisches Landesamt für Umwelt"*^9^ comprises data which mainly originate from long-term species and biotope monitoring programmes run by Bavarian state agencies. They are mostly categorised as observations and calculated to receive geographic accuracy, such as the CEBA quadrant grid data. The single information is evaluated according to name concept and taxonomic reliability.*^10^ The FSG occurrences from state-run survey studies (currently 123,439 of the 361,173) are an integral part of this BFL data source.

As of February 2022, both FSG sources together provided 361,173 records. The occurrence area was calculated by GIS shapes of the Bavarian Forest region. The shapes were deposited in the DWB cloud repository*^11^ for later reuse. The observations refer to more than 2,146 taxonomic names. After evaluation by local taxon experts and manual assignment, there were 1,381 taxa with taxon concepts accepted for the study area and agreed amongst the FSG partners (see Table 1).

### Design description

The data pipelines and other IT services established to achieve the objectives.

**Technical concepts of Pladias**

The Pladias software infrastructure is a result of the specific situation in the Czech Republic, where dozens of individual providers of data exist. These providers have to (for legislative reasons) or want to keep internal primary evidence of occurrence data on their own. Although all partners involved are aware of the need to share biodiversity information, there is no support for merging the databases into a single entity. Pladias, therefore, acts also as the primary source of data (directly uploaded by users), but most of the data are secondary, taken from partner institutions. For those records that are downloaded, we provide feedback to the primary data provider on expert validations, but it is at their discretion as to how they handle them.

The basis of the infrastructure is a relational database (= RDBMS; Relational Database Management System) PostgreSQL with PostGIS extension allowing us to process spatial data. A description of the database information model is provided by Novotný et al. (2022). Professional botanists use a web application*^12^ written in Java, the public having read-only access to the data provided by a web portal*^13^written in PHP.

Pladias does not offer standardised APIs; rather, exports are performed manually by direct SQL querying of the database or by database views. It also does not have an interface compliant to the GBIF network, mainly because of the already-mentioned large fragmentation of the licensing conditions of the different data providers. Cooperation with the Bavarian partners thus represents the first integration effort beyond the borders of the Czech Republic.

**Technical concepts of the BFL instance of DWB**

The modularised Diversity Workbench (= DWB) represents a freely-available open source tool suite for the management of life and environmental sciences data (see description in biotools*^14^and in GFBio*^15^). The tools may be adapted to handle different kinds of bio- and geodiversity data, taxonomies and terminologies and facilitates the processing of such data and information. The DWB is set up on a system of xml-enabled SQL relational databases. Rich clients are mostly installed as desktop applications. They might be run as an intranet solution and provide direct interoperability with clients of interlinked databases including cloud services. DWB installations are flexible in the management of highly-structured data, enable sophisticated user administration and allow for user-adapted data entry and data exchange.

The BFL instance of DWB currently consists of installations of the modules *DiversityCollection* and *DiversityTaxonNames* with supportive data collections in *DiversityAgents*, *DiversitySamplingPlots* and the project management undertaken in *DiversityProjects*. The dynamic access to (cloud-based) web services of the DWB user community, as well as to external data resources, is realised. With that wider concept, the Bayernflora initiative follows the Linked Data approach as visualised by Triebel et al. (2012). The scheme and model of the virtual working environment follows the FAIR principles of the digital object architecture regarding the occurrence presence/absence event as a core (see Harjes et al. 2020, Lannom et al. 2020). The BFL design is optimised to allow data aggregation and data publication of occurrence data via various service endpoints partly compliant with the GBIF network and other networks (see*^16^). For visualisation in the BIB data portal,*^6^ a kind of "taxonomic intelligence" (Patterson et al. 2008) with management of persistent identifiers (PIDs) for each kind of taxon name with indication of categories (e.g. currently accepted name, basionym, species name) and of relations to other names was set up (e.g. classifications, synonymy, vernacular names) (in *DiversityTaxonNames*). Furthermore, the BFL taxon name experts and various editors curate the indicator systems, for example, for floristic status (Ruff et al. 2019), for biology categories like those of Ellenberg's indicator values (EIVs) and for species conservation status (Red List status), as well as the vernacular names as used in various Bavarian regions. The service and software providers at the SNSB align their technical services to evolving German research data infrastructures like NFDI and GFBio e.V. to ensure technical sustainability.

**Data repositories, interoperability and data policies**

There are major differences in interoperability and data policies between the platforms of Pladias and the data repository of the Flora of Bavaria initiative. The data management strategy and agreed policies, as well as guidelines for data publication in the Bavarian part of the FSG project, are those of the Flora of Bavaria initiative*^17^. The reason for that is that the Bayernflora experts rely on a common Bayernflora instance and repository of the DWB virtual management platform (see above). DWB with its Bayernflora instance is mainly a RDBMS that has a sophisticated, but sustainable infrastructure for internal data curation processes, data interoperability, data export, archiving and format standardisation. There are several BioCASe guided data publications as zip-archives in the data exchange standard ABCD 2.0 and ABCD 2.1*^18^ with metadata information agreed amongst and suitable for science communities like GBIF and GFBio/ NFDI4Biodiversity (see Holetschek et al. 2012, Fichtmueller et al. 2019 and GFBio overview*^19^). Finally, both main stakeholders of the initiative, i.e. the volunteer expert community (AG Flora von Bayern) and the Bavarian Environment Agency (Bayerisches Landesamt für Umwelt), each act as its own GBIF dataset provider, as well as its own GFBio service endpoint (--> info on data content under "Flora of Bavaria ─ occurrence data online"*^8^ and "Floristic records from survey studies of the Bayerisches Landesamt für Umwelt"*^9^) (see Weiss et al. 2018). With that design, each single ABCD-mediated occurrence record is licensed with a common data policy and published under a CC-BY licence model as strongly recommended by the Global Biodiversity Information Facility (GBIF)*^20^

One of the primary objectives of Pladias was to provide Czech botanists with a tool for validation of occurrence data and creation of the basis for the forthcoming atlas of vascular plant distribution in the Czech Republic. For this reason, it contains a system of roles, taxon-user expert associations and a number of tools that allow us to classify the validity of records or communicate during the preparation of data for map publication. These mechanisms are based on the needs of the local community and, although by their nature they correspond to international standards, they were not created *a priori* out of an ambition to meet the requirements of the standards, but the requirements of the community in relation to the nature of the data available.

**Challenges of different taxon reference lists, dynamic taxon concepts and persistent identifiers**

Although the Czech Republic and Germany are neighbouring countries and share almost the same flora and vegetation in the region of interest, the concept of critical groups differs. This is caused by the deviating regional view on the dynamics of local plant populations, on major trends in modern systematics, changing taxon concepts and the setting of different research focus, as well as nature conservation focus on single plant groups of interest.

The Bayernflora DWB management instance uses the independent reference checklists and taxonomies of Lippert and Meierott (2014), Lippert and Meierott (2018) and their nomenclature and taxonomy concept updates. The Bavarian Taxonomic Reference list is curated by Diewald & Ahlmer (2016 onwards*^21^) using an independent *DiversityTaxonNames* (DWB-DTN) RDBMS instance (for the information model, see Hagedorn et al. 2019) with persistent BFL TaxRef IDs. The name backbone is presented via mediawiki website*^21^ with a download option and via dynamic access through the BIB regional PHP data portal*^6^. It is continuously discussed amongst the Flora of Bavaria expert team reflecting current concepts, for example, as promoted by Müller et al. (2021).

Pladias is using the reference monograph of Kaplan (2019) with onward curation by Kaplan, Štech & Danihelka, leading to single authoritative taxon reference lists in which each record is assigned to just one taxon. However, new relevant taxonomic treatments are included continuously. It does not use persistent identifiers other than the pure database level IDs, i.e. the model situation of just correcting a typo in a scientific name does not lead to a new ID (Novotný et al. 2022).

For comparison of the taxa from both checklists, a taxon converter with two parts was set up and curated by Štech & Diewald *^22^, ^23^. The different taxon concepts and deviating hierarchical classification concepts used in the two source databases do not allow for cardinality other than many-to-many. This has resulted in the introduction of a third taxonomy (in addition to Czech and Bavarian), to which we refer as FSG agreed (consensus) taxonomy with FSG taxa. Table 2 shows the background of the FSG taxon *Aconitumplicatum* as an example. This two-part converter is the cornerstone of the taxon concept interoperability and must be updated from time to time as both source databases are continuously and independently evolving in their taxonomic concepts. Its current version can be downloaded on the project website*^22^, ^23^.

**Challenges of different floristic status systems, validity/reliability and origin status systems and basis of record systems**

Each Pladias record holds one of four reliability statuses (reliable/uncertain/erroneous/not yet revised), one origin status (native/non-native/planted/not set) and the herbarium origin status (true/false) (see Wild et al. 2019).

The DWB BFL occurrences data sources, as far as published as ABCD2.1 XML zip-archives (BFLportal01, BFLportal04), are per definition categorised as validated, i.e. reliable sensu Pladias. The non-validated DWB BFL data records are not published via ABCD2.1, but stored in the internal RDMS instance. Each single "*in situ*" occurrence record has either a floristic status category assigned by the observer and/or a processed floristic status category assigned by a later editor. The procedure is explained in the BFL Wiki page on floristic status*^24^ with references to Pladias. In addition, each record is categorised according to a controlled vocabulary including "PreservedSpecimen", "HumanObservation" and "Literature" (similar to Czech herbarium origin status, see GFBio/ GBIF RecordBasis terms*^25^).

The translation of the BFL floristic status system with 13 status categories to the Pladias origin status system and to the status categories "native", "introduced" and "cultivated", as defined by the TDWG pre-standard POSS (World Conservation Monitoring Centre 1995), is shown in Table 3. With this pre-work, all data sources are well prepared to deliver agreed cross-border origin status, herbarium origin and floristic status concepts in the near future (for Bayernflora data sources documentation, see Ruff et al. 2019).

**Challenges of establishing agreed data pipelines**

The aim of the integration effort was to put in place a functioning mechanism for batch sharing of cross-border data and data sources. There were several reasons why we were not striving for a complete continuous process. One is that both countries have established independent data mobilisation, integration and publication processes ensuring sustainable and quality-controlled data sources for both countries, Czech Republic and Bavaria. Another one is the dynamics of taxonomic concepts and classifications as explained above and which is different in both countries. Assessing the compatibility of currently-accepted taxon concepts is a matter of professional review which is the most important phase in the data-sharing process and cannot be done other than by discussion of experts from both countries. An agreed dynamic data pipeline as a result is also challenging because of the tens of millions of BFL and Pladias records to be handled and the required functional stability of such a network. The target result is, therefore, a functional infrastructure that allows experts to quality-check, align and map data sources, come across trilingual aspects and transform data towards the FSG target system.

We also have to emphasise the question of the primary storage of converted data. Pladias cannot store data from Bavaria as it has a deeply embedded system constraint on a fixed polygon. The DWB instance of the BFL also cannot store Czech data as it does not meet its thematic focus and licensing requirements. Furthermore, the aggregated data is bound to a compromise FSG checklist, which again prevents its smooth incorporation into one of the participating systems. It was, therefore, necessary to create a separate data-linking technical service, running at South Bohemian University, which has connectivity to both partners and handles the integration processes.

**Functional infrastructure step by step**

***A. Data generation and integration***

**Step 1.** Occurrences for the FSG project from the Pladias project and external resources.

Occurrence records for the FSG project are imported into Pladias by individual users in the form of a standardised MS Excel spreadsheet. In addition to the floristic record, the user also indicates the licence of the record and its assignment to the specific project, like the FSG project, within which the data were created/are published. The data are created either as a direct result of floristic research or are excerpted from herbarium sheets, publications or come through separate import processes from data providers.

The first check of occurrence status validity is done by automatic mechanisms (e.g. compliance with the specified municipality polygon). Subsequently, the assigned auditors can indicate the reliability of the record (for reliability status system, see above).

**Step 2.** Occurrences for the FSG project by *DiversityMobile* and CSV files from FSG partners and the BFL project.

**Figure 4.**
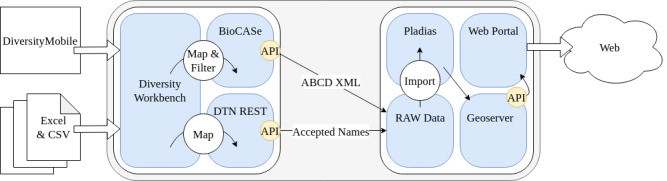
Data generation and mobilisation: Diversity Workbench (DWB) to Pladias.

The FSG partners from Bavaria routinely used a windows phone app *DiversityMobile* and workflow as described by Jablonski et al. (2009) for data generation. By using GPS-enabled smartphones*^14^, ^26^, ^27^, the FSG project mobilised more than 28,700 georeferenced records with WGS84 coordinates in the time span between 2019 and 2022 from the area of interest. The *DiversityMobile* app implements a direct data exchange with a Diversity Workbench instance running *DiversityCollection*, *DiversityProjects* and *DiversityTaxonNames* at the SNSB (see Fig. 4). Few FSG records were contributed via standardised MS Excel spreadsheets and CSV.

**Step 3.** Occurrence data storage in DWB, provision via ABCD XML and customisation for Pladias.

The BFL occurrence data in DWB are long-term curated according the DWB schema explained under "Technical concepts of the Bayernflora (BFL) instance of DWB" and "Data repositories, interoperability and data policies" (Fig. 4). The export of the BFL occurrence data and provision, for example, for Pladias, is done regularly every six months. It starts with transformation of data from the DWB DiversityCollection RDBMS (for information model, see Weiss et al. 2016) into the community standard ABCD, which is done by the Python-based BioCASe Provider Software. The two generated ABCD2.1 XML zip-archives (BFLportal01, BFLportal04)*^28^ include a persistent BFL TaxRef taxon-ID for each record which might be pointing to another BFL TaxRef taxon-ID that might be categorised as an accepted name, as well as a BFL synonym or BFL basionym. The ABCD 2.1 elements*^18^ used by an AddOn ABCD_BayernFlora are, amongst others, from the node "/*Identification/References/Reference*", specifically the following elements:

- "*ReferenceGUID*",
- "*TitleCitation*",
- "*CitationDetail*",
- "*URI*".

The initial technical connection between the dynamic DTN BFL backbone and the open access publication of the two BFL data sources as ABCD2.1 XML zip-archives (BFLportal01, BFLportal04) is regarded as a first benefit of database integration and allows for repeatable independent steps for the unification of a cross-border nomenclatural and systematic view of biodiversity.

**Figure 5.**
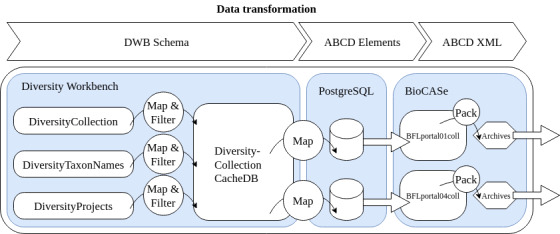
Transformation DWB occurrence data, XML archives.

The files prepared in this way are then imported into the dedicated FSG database. For this purpose, we developed a custom XSLT template suitable for efficient conversion of a reduced set of ABCD fields into SQL "INSERT" queries, leading to one SQL file from each of the packages of BFLportal01coll*^29^and BFLportal04coll*^30^ (Fig. 5). These technically converted data (RAW data) are imported into the Pladias project database (Fig. 4). Each data source (XML ABCD 2.1) in the SNSB BioCASe installation*^28^ represents a DWB cache database mapped and filtered to one or more conceptual schemas (like Darwin Core or ABCD 2.0, ABCD 2.1, LIDO; for schema descriptions, see GFBio overview*^19^).

**Step 4.** Spatial criteria postprocessing.

**Figure 6.**
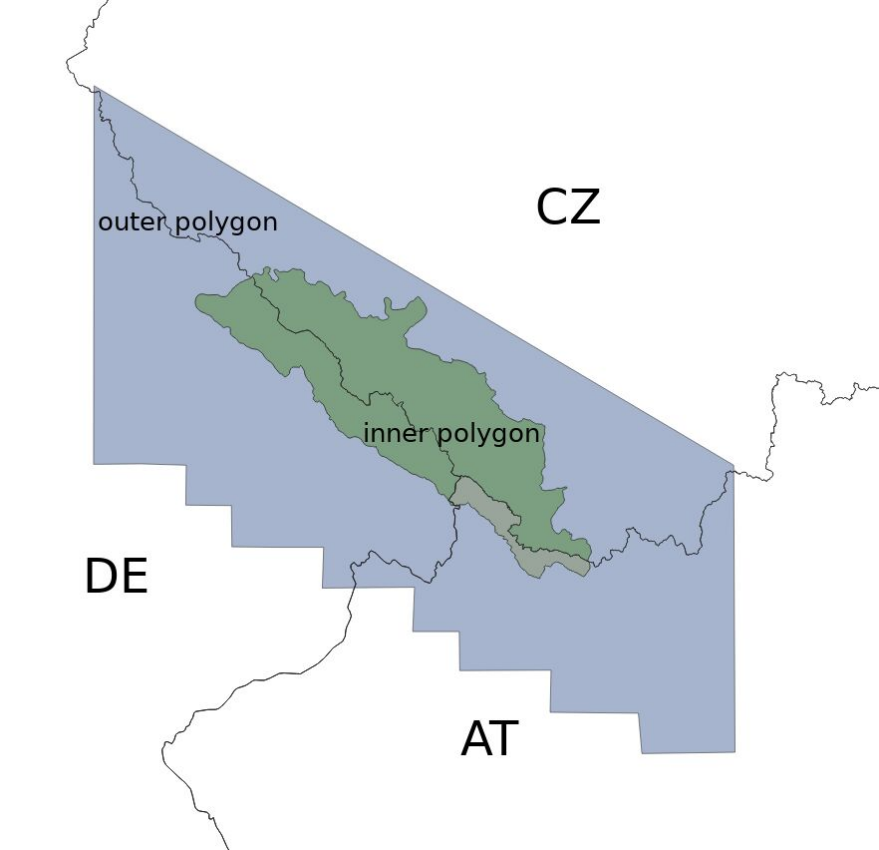
Area of Bohemian Forest. CZ = Czech Republic, DE = Germany/Bayern, AT = Austria. Blue filled polygon represents the outer polygon serving as the pool of candidate taxa for the study area. Green filled polygon's records are used for integration. Small grey part belongs to Austria and is not fully covered by current infrastructure.

The imported data may cover an area larger than necessary. Therefore, they are intersected against two polygons: i) the outer (study area + the surrounding strip of lower altitude landscape, which is a source of potential future increase in the biodiversity of the core area) and ii) the inner (= study area, the Bohemian Forest), see Fig. 6. Only records fitting the inner polygon are post-proccessed and used as final data for the FSG project. The outer polygon serves as a control check for taxa occurring nearby or candidate taxa which might be potentially found in the study area in the future. It includes additional data which are informative for the needs of the curators using the taxon converter.

***B. Data transformation***

**Step 5.** Processing of BFL taxon reference list.

**Figure 7.**
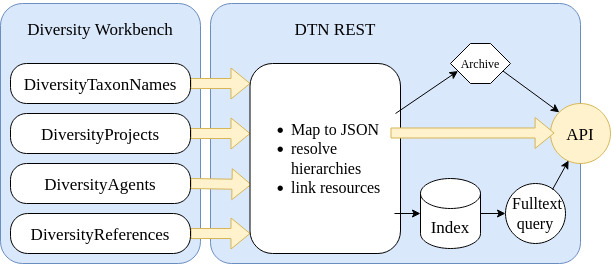
Transformation of DWB taxon names and classification data, JSON REST web service with API.

Raw imported records are post-processed using the DTN REST Web service (Seifert et al. 2015) which is optimised to organise a machine-usable direct access to persistent BFL TaxRef taxon IDs (using a SQL server function, DP project ID: 1129). The service has several endpoints for GET requests, amongst others, retrieving the BFL accepted name and Tax-Ref PID, synonym/basionym names PIDs, as well as related information, like scientific and vernacular names (Fig. 7).

Amongst others, the following DTN REST Web service end points are used for processing taxon names:

1. First, we resolve the accepted taxon ID by using the endpoint http://services.snsb.info/DTNtaxonlists/rest/v0.1/names/DiversityTaxonNames_Plants/__NAME___/acceptednameid/.
2. Second, for these identificators, we resolve the scientific names by using the endpoint http://services.snsb.info/DTNtaxonlists/rest/v0.1/names/DiversityTaxonNames_Plants/__ID__.

From this point on, we are able to link DWB and Pladias taxa via a two-part taxon converter*^22^, ^23^ and work with the consensus (compromise) concept –⁠ the FSG taxa.

**Step 6.** Postprocessing of floristic and origin status and other record metadata.

The different approaches for describing nature conservation information, defining basis of record categories and floristic status are apparent after the records are linked to the agreed taxa. Both database systems have well-documented concepts to dynamically assign floristic status, occurrence validity, origin status (native/non-native/planted/unknown) or herbarium origin status and basis of record categories, respectively. We developed several mappings of categories for a more or less wide interoperability of plant occurrence and status categories (see Table 3), but most of them are nowadays stored only for evidence. Future optimisation of the Bohemian Forest portal will deal with this issue in more detail.

***C. Data publishing***

**Step 7** Data portal with Flora of the Bohemian Forest (FSG) taxon distribution maps.

Joined taxon distribution is presented on the FSG web portal in the form of dynamic maps composed by the OpenLayers library from a Web Map Service (WMS) provided by a local instance of Geoserver (for examples, see Fig. 2 and Fig. 3). Data are generalised on the level of CEBA quadrants (= TK25/4) and labelled by the highest reached reliablity status in each quadrant.

Both partner databases are specialised data repositories hosted by large institutions. They have fixed validation processes and data publishing pipelines, just as the FSG pipeline has a fixed update frequency. In order to be able to guarantee the timeliness of the presented maps, regardless of the dynamics of all involved components, the data portal includes an additional function for manually entering the quadrant-taxon reliability status not based on current data.

## Web location (URIs)

Homepage: https://www.florasilvaegabretae.eu/

## Technical specification

Platform: Pladias Service: Docker, Geoserver

Programming language: Pladias Service: XSLT, SQL, PHP

Operational system: Pladias Service: Linux

Interface language: Pladias Service: PHP, JS

Service endpoint: https://www.florasilvaegabretae.eu/

## Usage licence

### Usage licence

Creative Commons Public Domain Waiver (CC-Zero)

## Implementation

### Audience

The audience for the flora of the Bohemian Forest portal*^1^ is the cross-border civil society with strong emphasis for nature and habitat conservation. The engaged persons have at least basic knowledge of native plants of the Bohemian Forest combined with strong interest in nature, biogeography and botany. The portal is realised with a trilingual interface in Czech, German and English languages with common lists of taxa (vernacular names, scientific names)*^31^, ^32^ and commentaries about distribution pattern, occurrence history and ecology of species in the study area and plant images taken in the region. The main stakeholders might include nature park and forest rangers, global change activists, plant and nature enthusiasts, citizen scientists, vegetation experts and students, professional botanists, farmers, foresters, ecologists, nature conservationists and persons interested in ethnobotany and traditions.

The implementation of the described infrastructure brought the cross-border local communities and the regional experiences together and supports the intercultural exchange with emphasis. At the same time, the new technical services rely on long-term exchange of digital information along established data pipelines in the Czech Republic and Bavaria. In the future, it would be very useful to expand the network and include the Austrian finds in the database for complete coverage of the study area.

## Additional information

**Funding**

Since 1990, the European Union is supporting territorial cooperations between regions and cities through the programme "Interreg" of the European Regional Development Fund (ERDF). The ERDF focuses its investments on projects on infrastructure, cooperation between public utilities, collaborative actions of companies or activities in the field of environment protection, education, land-use planning and culture. The fifth funding period of the programme, i.e. ETC "European Territorial Cooperation" INTERREG V, has been set up from 2014 to 2020.

The project "Flora of the Bohemian Forest (Flora Silvae Gabretae, FSG)" is an action to establish a cross-border cooperation between the Free State of Bavaria and the Czech Republic (Ziel ETZ, INTERREG A; EU-project number 216). The applicable project period is from 01-01-2019 to 30-06-2022. For Bavaria, the action is managed by the Government of Lower Bavaria. The focus on nature conservation, protection of the environment and planning of resource efficiency.

The financial support, called “Ziel ETZ”, is approx. 1 million € (in total). There is a co-financing of 15% (from the partner country/region, public support).

Additional funding is provided by the Bavarian En­vi­ron­ment Agen­cy (LfU) and the German Research Foundation (DFG) with GFBio and NFDI4Biodiversity, a consortium of the German National Research Data Infrastructure (NFDI).

## Figures and Tables

**Table 1.** Overview of data sources for the Bohemian Forest region used in the cross border cooperation of the Flora Silvae Gabretae (FSG) project (status Dec 2021).

	**Area (covered by technical platform, km^2^)**	**Area (covered by FSG Project, km^2^)**	**Number of FSG Occurrence Records (polygon)**	**Number of FSG Occurrence Records under CC-BY licence**	**Number of High Quality Occurrence Records (GIS referenced or exact locality indicated)**	**Number of Taxa (taxon concept agreed amongst FSG partners)**
Pladias	78,871 km^2^	1,846 km^2^	13,833	3,263	576,673	1,615
Flora von Bayern	70,550 km^2^	918 km^2^	361,173	361,173	176,161	1,381
Austria	unknown	231 km^2^	unknown	unknown	unknown	unknown

**Table 2.** Example of many-to-many records in taxa mapping between Pladias and Bayernflora (BFL) taxa, resulting in compromise FSG taxon *Aconitumplicatum*.

**Pladias taxa**	**FSG taxon**	**BFL taxa with BFL TaxRef ID**
	*Aconitumplicatum* [ID_FSG = 14]	Aconitumnapellussubsp.lusitanicum Rouy [ID_BFL TaxRef = 20111]
*Aconitumnapellus* agg. [ID_PLADIAS = 1238]	*Aconitumplicatum* [ID_FSG = 14]	*Aconitumnapellus* L. s. l. [ID_BFL TaxRef = 52]
*Aconitumplicatum* [ID_PLADIAS = 1444]	*Aconitumplicatum* [ID_FSG = 14]	AconitumnapellusL.subsp.napellus [ID_BFL TaxRef = 6539]
	*Aconitumplicatum* [ID_FSG = 14]	*Aconitumplicatum* Köhler ex Rchb.[ID_BFL TaxRef = 14276]

**Table 3.** Assignment of categories of BFL/BIB floristic "in situ" status for valid present occurrences to the Pladias taxon origin categories and POSS pre-standard categories (for BFL/BIB status definitions see,*^24^ for POSS status definitions, see World Conservation Monitoring Centre 1995).

**BFL/BIB floristic "in situ" status category**	**Pladias taxon origin status category**	**POSS native status category**	**POSS introduced status category**	**POSS cultivated status category**
indigenous (I)	native	native	not introduced	not cultivated
"normal status" (*)	native	assumed to be native	not introduced	not cultivated
established (E)	non-native	not native	introduced	not cultivated
permanently established (D)	without equivalent	doubtfully native	assumed to be introduced	not cultivated
casual (U)	without equivalent	not native	introduced	not cultivated
tendency towards establishment (T)	non-native	not native	introduced	not cultivated
re-introduced / naturally casual (W)	without equivalent	native	introduced	not cultivated
cultivated (K)	planted	not native	none of the above	cultivated outdoors
synanthropic (S)	without equivalent	not native	none of the above	no information
deliberately introduced (A)	planted	not native	none of the above	cultivated outdoors
culture relic (R)	planted	not native	none of the above	cultivated outdoors
status completely unclear (?)	without equivalent	no information	no information	no information
dubious if native (Z)	without equivalent	doubtfully native	doubtfully introduced	not cultivated

## Footnotes

*1
https://www.florasilvaegabretae.eu/

*2
https://wiki.bayernflora.de/web/Geschichte

*3
https://wiki.bayernflora.de/web/Koordinationsstelle_f%C3%BCr_Florenschutz_in_Bayern

*4
https://wiki.bayernflora.de/web/Aktualisierung_der_Roten_Liste_der_Gef%C3%A4%C3%9Fpflanzen_Bayerns

*5
https://wiki.bayernflora.de/web/Flora_Silvae_Gabretae_%E2%80%93_Flora_des_B%C3%B6hmerwaldes_%E2%80%93_Kv%C4%9Btena_%C5%A0umavy

*6
https://daten.bayernflora.de

*7
https://diversityworkbench.net

*8
https://wiki.bayernflora.de/web/Flora_of_Bavaria_%E2%94%80_occurrence_data_online

*9
https://wiki.bayernflora.de/web/Floristic_records_from_survey_studies_of_the_Bayerisches_Landesamt_f%C3%BCr_Umwelt

*10
https://wiki.bayernflora.de/web/Daten_der_Biotopkartierung_des_Bayerischen_Landesamts_f%C3%BCr_Umwelt_f%C3%BCr_die_Flora_von_Bayern

*11
https://diversityworkbench.net/Portal/DWB_cloud_services

*12
https://pladias.ibot.cas.cz/

*13
https://pladias.cz/

*14
https://bio.tools/t?page=1&q=DWB&sort=score

*15
https://www.gfbio.org/tools

*16
https://wiki.bayernflora.de/web/Datenfluss_%E2%94%80_Flora_von_Bayern

*17
https://wiki.bayernflora.de/web/Dokumente#Richtlinien_zum_Umgang_mit_Daten

*18
https://abcd.tdwg.org/xml/documentation/ABCD_2.1/

*19
https://gfbio.biowikifarm.net/wiki/Data_exchange_standards,_protocols_and_formats_relevant_for_the_collection_data_domain_within_the_GFBio_network

*20
https://www.gbif.org/terms

*21
https://wiki.bayernflora.de/web/Taxonomische_Referenzliste_der_Gef%C3%A4%C3%9Fpflanzen_Bayerns

*22
https://www.florasilvaegabretae.eu/download/convertor-pladias

*23
https://www.florasilvaegabretae.eu/download/convertor-bayern

*24
https://wiki.bayernflora.de/web/Flora_of_Bavaria:_floristic_status

*25
https://gfbio.biowikifarm.net/wiki/RecordBasis

*26
https://wiki.bayernflora.de/web/Datenbank-Anwendungssoftware_zur_Eingabe_von_BFL-Daten

*27
http://www.diversitymobile.net/wiki/DiversityMobile_Applications

*28
https://biocase.snsb.info/wrapper/

*29
https://biocase.snsb.info/wrapper/dsa_info.cgi?dsa=BFLportal01coll

*30
https://biocase.snsb.info/wrapper/dsa_info.cgi?dsa=BFLportal04coll

*31
https://www.florasilvaegabretae.eu/homepage/taxa

*32
https://www.florasilvaegabretae.eu/de/homepage/taxa
